# 595. Recovery of *Clostridioides difficile* Isolates from Cary-Blair Transport Medium Compared to No Transport Medium

**DOI:** 10.1093/ofid/ofad500.662

**Published:** 2023-11-27

**Authors:** Andrew M Skinner, Laurica A Petrella, Susan P Sambol, Stuart Johnson, Christopher A Czaja, Helen Johnston, Elizabeth Basiliere, Robin Dhonau, Lauren C Korhonen, Ashley Paulick, Michelle Adamczyk, Alice Guh, Amy Gargis, Dale N Gerding

**Affiliations:** University of Utah, Holladay, Utah; Edward Hines Jr VA Hospital, Hines, Illinois; Edward Hines Jr. VA Hospital, Hines, Illinois; Hines VA Hospital and Loyola University Medical Center, Hines, Illinois; Colorado Department of Public Health and Environment, Denver, Colorado; Colorado Department of Public Health, Denver, Colorado; Colorado Department of Public Health and Environment, Denver, Colorado; Georgia Emerging Infections Program, Decatur, GA; Emory University School of Medicine, Atlanta, GA; Atlanta Veterans Affairs Medical Center, Decatur, GA, Brookhaven, Georgia; CDC, Atlanta, Georgia; Centers for Disease Control and Prevention, Atlanta, GA; CDC, Atlanta, Georgia; CDC, Atlanta, Georgia; Centers for Disease Control & Prevnetion, Atlanta, GA; Edward Hines, Jr. Veterans Affairs Hospital, Hines, Illinois

## Abstract

**Background:**

Surveillance of *C. difficile* infection (CDI), one of the most common U.S. healthcare-associated infections, is essential to inform public health response, characterize strains causing disease, and describe changes in strain prevalence over time. The U.S. CDC’s Emerging Infections Program (EIP) has conducted active CDI surveillance, including culturing of *C. difficile* from a convenience sample of stool samples from cases, since 2009. Cary-Blair transport medium (CBTM) is used by some clinical laboratories for stool testing, but little is known about its impact on the recovery of *C. difficile* from culture.

**Methods:**

We performed a cross-sectional study of the recovery of *C. difficile* isolates from *C. difficile* positive stools received 10/2021 – 10/2022 from Colorado and Georgia EIP sites. Stools were collected and stored within CBTM or within no transport medium per site protocols and subsequently frozen. Frozen samples were thawed and inoculated on taurocholate-cycloserine-cefoxitin-fructose agar plates (TCCFA) for recovery. Specimens from which *C. difficile* was not recovered on TCCFA plates were subjected to alcohol shock to enhance the recovery rates. We compared the recovery rates of *C. difficile* from stools in CBTM and stools in no transport medium.

**Results:**

We cultured 825 stools from Colorado and Georgia, recovering *C. difficile* from 81.5% of stools. Overall, 412 (49.9%) stools were in CBTM and 413 (50.1%) were stools in no transport medium. Of those with known testing data, 410/410 (100%) CBTM stools vs 371/407 (91.2%) non-CBTM stools were *C. difficile* positive by PCR test (p< 0.01). Colorado stools accounted for 78% of the CBTM specimens. We recovered *C. difficile* on TCCFA plates from 59% (244/412) of stools in CBTM compared to 84% (348/413) in no transport medium (p< 0.01). After including alcohol shock results, *C. difficile* was recovered from 72% of CBTM stools, and 91% from stools in no medium (p< 0.01).

**Conclusion:**

Cary-Blair transport medium may limit the recovery of *C. difficile* from culture and impact the ability to detect emerging strains. A large parallel study is needed to determine if these findings are reproducible and if stool collection methods should be standardized to maximize the recoverability of *C. difficile* by culture.

**Disclosures:**

**Andrew M. Skinner, MD**, Academy for Continued Healthcare Learning: Honoraria|American Society of Healthcare Pharmacists: Honoraria|Ferring Pharmaceuticals: Honoraria|MJH Life Sciences: Honoraria **Dale N. Gerding, MD**, Destiny Pharma,: Advisor/Consultant|Sebela Pharma: Advisor/Consultant

